# Surgical Outcomes of Post-myocardial Infarction Ventricular Septal Defect/Rupture: A Systematic Review and Meta-Analysis

**DOI:** 10.7759/cureus.44135

**Published:** 2023-08-25

**Authors:** Soumitra Ghosh, Vikram Halder, Amit Mishra, Maruti Haranal, Pankaj Aggarwal, Parag Barwad, Harkant Singh, Shyam Thingnam, Vidur Bansal

**Affiliations:** 1 Cardiology, Post Graduate Institute of Medical Education & Research, Chandigarh, IND; 2 Cardiothoracic Surgery, U. N. Mehta Institute of Cardiology & Research Centre, Ahmedabad, IND; 3 Cardiothoracic Surgery, Post Graduate Institute of Medical Education and Research, Chandigarh, IND; 4 Cardiology, Post Graduate Institute of Medical Education and Research, Chandigarh, IND

**Keywords:** cardiogenic shock, mortality, surgical outcomes, ventricular septal defect, myocardial infarction

## Abstract

Ventricular septal defect (VSD) is a catastrophic acute myocardial infarction (MI) complication. Despite a significant reduction in the prevalence of post-MI VSD with the advancement of surgical techniques, it is still considered fatal with a high mortality rate. The trends in the clinical outcomes of patients with post-MI VSD show discretion due to the complexity of the disease. Therefore, the present analysis aimed to evaluate the surgical outcomes and associated risks in the patients of post-MI VSD. A thorough literature survey resulted in 40 studies of our interest. The pooled proportion of differential variables, including the incidence of cardiogenic shock, 30-day survival, and overall mortality, were estimated using Bayesian hierarchical models. The risk difference was estimated for the location of MI and VSD and mortality in patients with coronary artery bypass graft (CABG). In addition, the heterogeneity tests for inconsistency and publication biases using Egger’s and Begg’s tests were also estimated. The analysis revealed a significant risk difference of 0.23 and 0.27 for the anterior vs. posterior location of MI and VSD, respectively. Further, the pooled proportion of 30-day survival and mortality was found to be 54.43% (95% credible interval (CI): 52.88-55.98%) and 48.22% (95% CI: 4-12.3%), respectively.

Moreover, the heterogeneity test revealed significant inconsistencies in all the datasets with *an I2 *index of >90% (*p*<0.0001). Lastly, the publication bias results suggested no evidence of asymmetry and small-study effects. Conclusively, the surgical management of post-MI VSD patients is considered beneficial; however, the outcomes signify its fatal behavior.

## Introduction and background

Ventricular septal defect (VSD) or ventricular septal rupture (VSR) is a serious complication of acute myocardial infarction (MI) [1]. During ventricular remodeling, VSD may occur within the initial insult of 3 days to two weeks post-MI. VSD or MI may be localized in the anterior or posterior wall or septum [1,2]. The concomitant risk factors of coronary artery disease include smoking, dyslipidemia, hypertension, diabetes mellitus, etc., which are found to be associated with post-MI VSD that may complicate the diseased condition [3-6]. However, the overall effects of risk factors on the clinical outcomes of the post-MI VSD remain obscure. Post-MI VSD is considered fatal due to the poor prognosis of the disease [1-4]. Surgery is considered the gold standard for the treatment of post-MI VSD [7]. Clinically, differential techniques such as patch closure, infarct exclusion technique, and modified infarct exclusion technique are used to correct post-MI VSD [8-10]. Disease recognition, hemodynamic stability, and surgical intervention are the key determinants for successfully managing the disease [11]. Current guidelines recommend early and urgent repair of the defect to manage hemodynamically unstable post-MI VSD patients [12]. Timing from MI to VSD and VSD to repair are highly controversial variables. Delay in the surgical repair may be associated with poor outcomes in unstable patients and may result in high mortality of patients awaiting surgical intervention in this group, while the outcome is better if operated after the acute episode [13]. With the introduction of differential therapies for post-MI VSD correction, the incidence of post-MI VSD has been greatly reduced to 0.2% from 3% in the past few decades [1,2,14]. Despite this, the surgical management of post-MI VSD is highly challenging and is associated with extremely poor outcomes such as postoperative low cardiac output syndrome, low 30-day survival rates after surgery, and high mortality rate [15]. Treatment of post-MI VSD without surgical interventions may result in >90% of mortality [15]. To date, there is limited knowledge on the overall effects of risk factors, location of VSD and MI, surgical intervention with or without coronary artery bypass graph (CABG), and preoperative intra-aortic balloon pump (IABP) on the clinical outcomes of patients with post-MI VSD. Based on this background, this meta-analysis reviewed the clinical and surgical experiences to establish the possible outcomes of post-MI VSD.

## Review

Methods

Search Tool

The guidelines of the Cochrane Handbook and Meta-analysis of Observational Studies in Epidemiology [16], prepared according to the Preferred Reporting Items for Systematic Reviews and Meta-Analyses (PRISMA) recommendations, were followed for this meta-analysis. A thorough literature search was performed through PubMed, Scopus, Cochrane, and Google Scholar databases with the following combinations: “ventricular septal defect,” “VSD,” “VSR,” “post myocardial infarction,” “post-MI VSD,” “surgical outcomes,” “post-MI VSR,” and “ventricular septal rupture”. The published studies in the English language from 1995 to 2020 were considered for meta-analysis. Case reports, expert opinions, literature reviews, editorials, and conference abstracts were excluded from the study.

Data Extraction

The following parameters were noted: age, gender, pre-procedures such as percutaneous coronary intervention (PCI), Qp: Qs and inotropes, ejection fraction and surgical procedures, timings of MI to VSD and VSD to repair in days, residual shunt and affected coronary artery. The analyzed parameters for the meta-analysis were: incidence of cardiogenic shock in PMI-VSD patients, anatomical location of MI, location of VSR, preoperative IABP and concomitant CABG, risk factors (prior myocardial infarction, diabetes mellitus, smoking, and dyslipidemia), 30-day survival, and overall mortality along with mortality associated with concomitant CABG, location of MI and location of VSR.

Statistical Analysis

The clinical and surgical outcomes data were retrieved from the selected publications or calculated after extracting the numeric data. Depending on the data, the pooled proportions of surgical outcomes and associated factors across studies were estimated from the exact number of patients with 95% credible intervals [CI] using the Bayesian hierarchical models or fixed-effects meta-regression of the natural logarithm of the risk difference was performed for comparative analysis. The results were depicted using forest plots. Further, heterogeneity tests for inconsistency (I2) levels and Egger’s and Begg’s tests for publication bias were performed across the selected publications. Publication bias is considered when deciding to publish a manuscript depending on statistically significant results. The statistical analysis was performed using MetaXL software.

Results

Search Results

**Figure 1:**
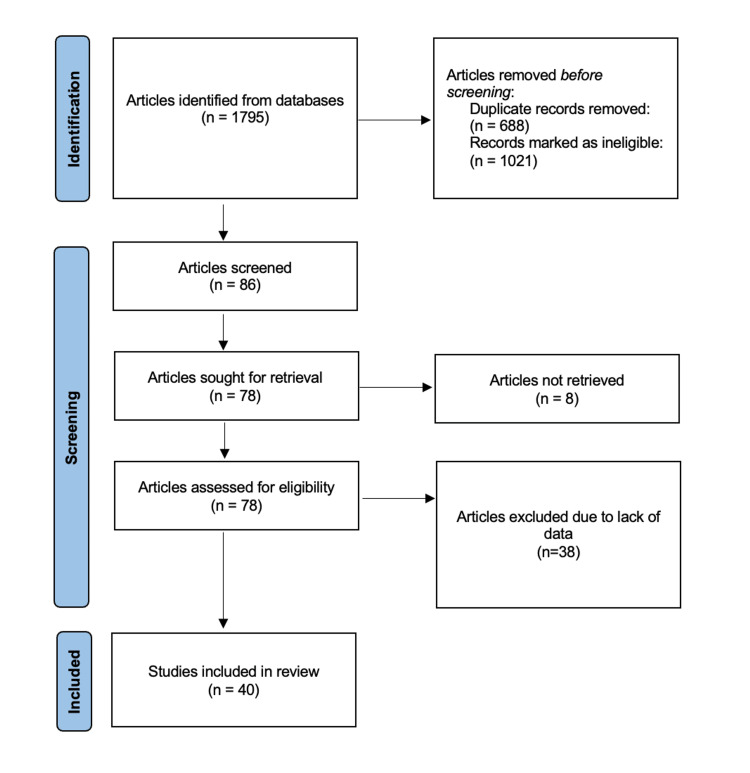
Consort flow diagram

Despite the declined incidence of post-MI VSD, the literature search resulted in 1795 articles; however, only 86 studies were identified as pertinent. Among the pertinent articles, 40 were considered for the final meta-analysis based on the data of interest (Figure 1). Table 1 summarizes the study characteristics and other variables specified in these publications. A total of 4,028 post-MI VSD patients with a mean age of 69.83 ± 8.2 years were included in the analysis. The proportion of males and females was 1.11:1 (38 studies). The average timing of MI to VSD diagnosis was 4.5 ± 3.3 days, while the average timing from VSD diagnosis to repair was 21.5 ± 40.1 days [17-44].

**Table 1:** Study characteristics and operative outcomes n = number of subjects, y = years, % = percentage, M = males, F = females, PCI= Percutaneous intervention

S. No.	Studies included	Data collection in years	Sample Size (n)	Mean Age (y)	Gender		Pre-procedures			Timing of VSD from MI in days	Timingfrom VSD to repair in days	Ejection fraction (%)	Follow-up (y)
					M (n)	F (n)	PCI (n)	Qp: Qs	Ionotropes (n)				
1	Heckle et al., 2020 [26]	2001-2014	126	69.8	73	53	-	1.26	-	3.5	92.5	-	-
2	Wiemers et al., 2012 [27]	2000-2008	10	65.3	5	5	3	-	6	3.5	18.12	52	3.4
3	Yam et al., 2012 [28]	1995-2012	40	69	16	24	17	-	28	2	3	55	5.2
4	Serpytis et al., 2014 [6]	1991-2007	41	67.5	15	26	-	-	-	-	39	55	-
5	Vondran et al., 2020 [22]	1994-2016	53	68.9	30	23	-	-	-	-	11.9	41.4	-
6	Isoda et al., 2012 [14]	2001-2010	7	70.9	3	4	-	3.68	-	-	4	-	4.1
7	Arnaoutakis et al., 2019 [23]	2008-2012	537	74	277	192	131	-	-	-	8.4	55	-
8	Aggarwal et al., 2018 [34]	2000-2014	21	66.4	15	6	4	-	17	-	-	-	-
9	Drobac et al., 1983 [35]	-	13	64	8	5	-	-	-	5	21.63	-	-
10	Sabiniewicz et al., 2017 [36]	2003-2016	20	70	11	9	16	1.5	-	-	182.6	38.5	2
11	Tang et al., 2015 [1]	2006-2013	11	67	4	7	6	3	-	1.5	18	44	2.5
12	Anderson et al., 1989 [2]	1980-1987	68	65.7	41	27	-	-	13	-	51	-	4.08
13	Labrousse et al., 2002 [3]	1971-2001	85	69.5	51	34	-	-	-	-	3.4	-	8.6
14	Rhydwen et al., 2002 [37]	1995-1999	29	68	21	8	-	-	29	5.5	1	-	-
15	Malhotra et al., 2017 [29]	2009-2014	40	61.65	26	14	-	-	-	3.2	6.2	-	2
16	Deja et al., 2000 [30]	1986-1998	110	65.6	69	41	-	-	54	5.6	9	-	-
17	Hamilton et al., 2017 [17]	2006-2016	30	71	22	8	-	-	-	12	16	-	-
18	Isoda et al., 2016 [18]	2001-2013	24	73.5	8	16	-	3.9	-	-	2	-	-
19	Landzberg & Lock, 1998 [38]	1990-1998	18	68	-	-	-	-	13	-	-	-	5
20	Gregoric et al., 2014 [11]		11	52	8	3	-	-	-	-	3.6	-	-
21	Cerin et al., 2003 [12]	1992-2000	58	73	29	29	-	2.6	-	4	15	40	-
22	Jeppson et al., 2005 [13]	1992-1998	189	69	119	70	-	-	-	4	1	-	2.4
23	Feneley et al., 1983 [19]	1972-1981	33	65	19	14	-	3.4	-	2.6	-	40	-
24	Becker et al., 1999 [39]		65	70.9	26	39	-	-	-	4.3	-	-	-
25	Crenshaw et al., 1999 [4]		84	72	36	48	-	-	-	0.9	3.1	40	-
26	Tai et al., 2018 [44]	2007-2017	96	66	55	41	-	-	-	-	-	50	-
27	Dagget et al., 1977 [7]	1968-1977	43	62	24	19	-	-	-	-	-	-	-
28	Menon et al., 2000 [21]		55	72	23	32	-	-	-	1	-	40	-
29	Trivedi et al., 2015 [40]	2006-2012	20	67	11	9	-	3.28	-	6	-	48	-
30	Zhang et al., 2017 [41]	2003-2015	15	63	6	9	2	1.8	-	-	-	48	1
31	Calvert et al., 2014 [43]	1997-2012	53	72	31	22	15	-	30	-	-	50	-
32	Barker et al., 2003 [31]	1997-2002	65	64	40	25	-	-	22	4.5	1.5	50	-
33	Moreyra et al., 2010 [33]	1990-2007	408	71	198	210	-	-	-	-	-	-	-
34	Sakaguchi et al., 2019 [32]	2008-2014	1397	74.1	671	726	508	-	-	-	-	-	-
35	Hirotani et al., 2002 [8]	1993-2000	9	73.8	5	4	-	-	2	1.9	4.1	43	4.8
36	Huang et al., 2015 [5]	1995-2013	47	68	28	19	17	3	-	-	-	46	-
37	Parachuri et al., 2019 [9]	2013-2018	32	-	-	-	-	-	-	-	-	-	-
38	Bayezid et al., 2005 [10]	1999-2004	4	57	4	0	-	-	-	14	1	30	2
39	Pradhan et al., 2018 [15]	2013-2016	51	63.8	26	25	-	-	-	4.9	-	42	-
40	Sugimoto et al., 2008 [42]	1996-2006	10	74	5	5	-	3.5	-	-	-	-	-

Location of MI and VSR

**Figure 2:**
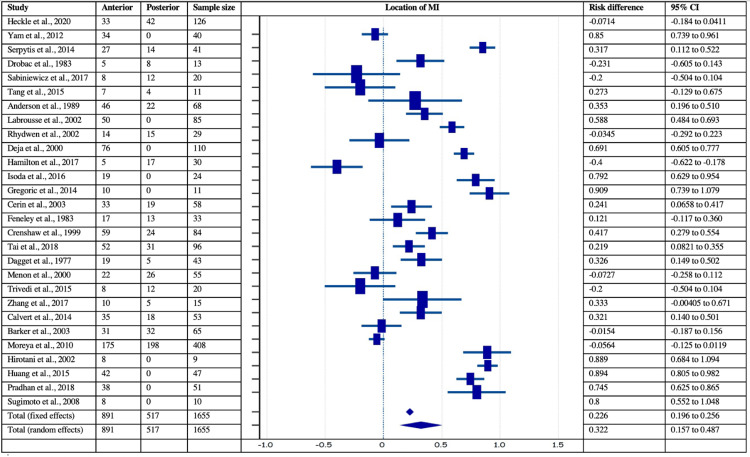
Forest plot showing risk difference with respect to location of myocardial infarction (MI) in post-MI VSD patients. Heckle et al., 2020 [26], Yam et al., 2012 [28], Serpytis et al., 2014 [6], Drobac et al., 1983 [35], Sabiniewicz et al., 2017 [36], Tang et al., 2015 [1], Anderson et al., 1989 [2], Labrousse et al., 2002 [3], Rhydwen et al., 2002 [37], Deja et al., 2000 [30], Hamilton et al., 2017 [17], Isoda et al., 2016 [18], Gregoric et al., 2014 [11], Cerin et al., 2003 [12], Feneley et al., 1983 [19], Becker et al., 1999 [39], Crenshaw et al., 1999 [4], Tai et al., 2018 [44], Dagget et al., 1977 [7], Menon et al., 2000 [21], Trivedi et al., 2015 [40], Zhang et al., 2017 [41], Calvert et al., 2014 [43], Barker et al., 2003 [31], Moreyra et al., 2010 [33], Hirotani et al., 2002 [8], Huang et al., 2015 [5], Pradhan et al., 2018 [15], Sugimoto et al., 2008 [42]. MI: myocardial infarction; VSD: Ventricular septal defect

Of the 40 identified studies, 28 with 1655 patients and 15 with 586 patients provided data for the location of MI and VSR, respectively. Among 1655 patients, the infarction was localized anteriorly in 891 (53.83%) patients and posteriorly in 764 (46.17%) patients (28 studies). The analysis revealed a significant risk difference of 0.23 in the anterior vs. posterior location of MI (z= 14.59, p<0.001, 95% CI = 0.20-0.26). The I2 index was estimated to be 97.07% (p<0.0001, 95% CI=96.44-97.59), which was significant. In addition, Egger’s (intercept = -1.93, 95% CI = -6.89-3.03, p = 0.43) and Begg’s (Kendall’s Tau =-0.231, p = 0.08) tests showed non-significant results, stating no evidence of publication biases concerning the location of MI. The forest plot of risk difference estimation for the location of MI is given in Figure 2.

**Figure 3:**
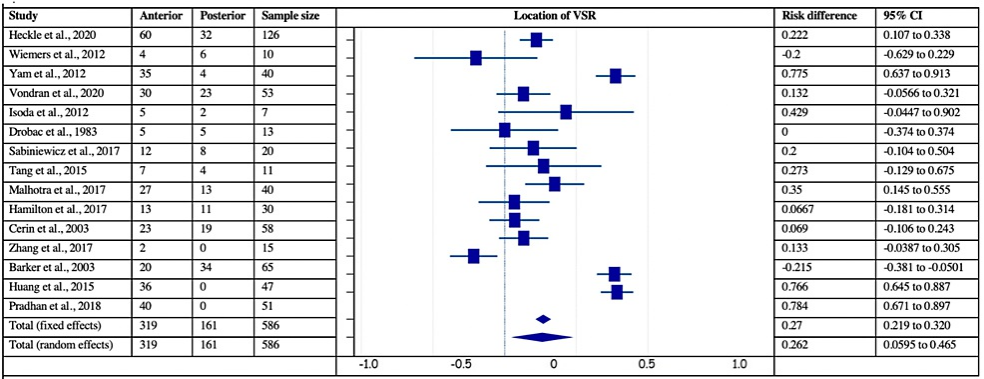
Forest plot showing risk difference with respect to location of ventricular septal rupture (VSR) in post-MI VSD patients Heckle et al., 2020 [26], Wiemers et al., 2012 [27], Yam et al., 2012 [28], Vondran et al., 2020 [22], Isoda et al., 2012 [14], Drobac et al., 1983 [35], Sabiniewicz et al., 2017 [36], Tang et al., 2015 [1],  Malhotra et al., 2017 [29],  Hamilton et al., 2017 [17], Cerin et al., 2003 [12], Zhang et al., 2017 [41], Barker et al., 2003 [31], Huang et al., 2015 [5], Pradhan et al., 2018 [15]. VSR: ventricular septal rupture; MI: myocardial infarction; VSD: Ventricular septal defect

Further, 15 studies reported VSD localization in the anterior wall of 319 (54.43%) patients and the posterior wall of 267 (45.57%). The risk difference of anterior vs. posterior location of VSD was estimated to be 0.27, which was significant (z= 10.52, p<0.001, 95% CI = 0.22-0.32). The heterogeneity tests revealed a significant I2 index of 94.39% (p<0.0001, 95% CI=92.19-95.96). In contrast, Egger’s (intercept = -4.32, 95% CI = -9.47-0.81, p = 0.09) and Begg’s (Kendall’s Tau = 0.047, p = 0.81) tests revealed no publication biases in specifying the location of VSD in the patients. The forest plot of risk difference estimation for the location of VSD is given in Figure 3.

Associated Risk Factors

**Figure 4:**
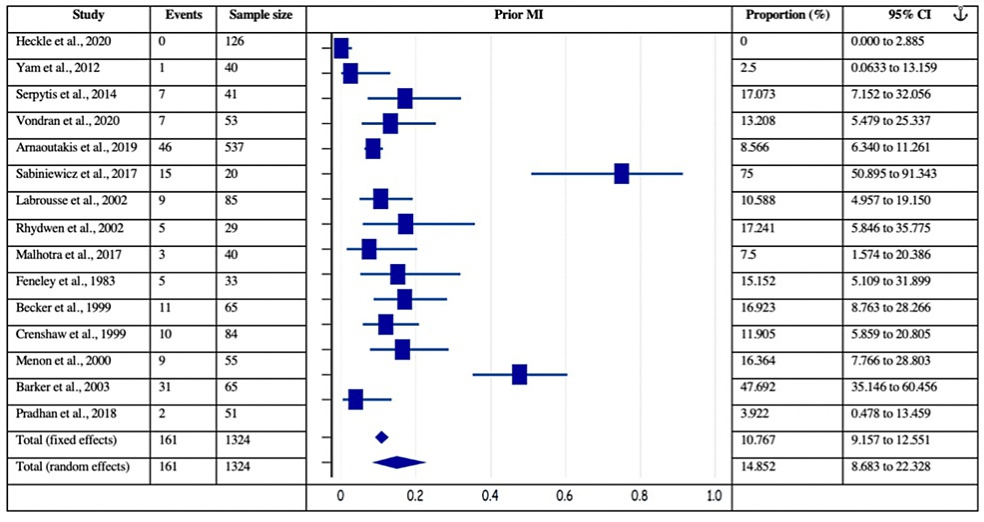
Forest plot showing prior MI as a risk factor associated with post-MI VSD. Heckle et al., 2020 [26], Yam et al., 2012 [28], Serpytis et al., 2014 [6], Vondran et al., 2020 [22], Arnaoutakis et al., 2019 [23], Sabiniewicz et al., 2017 [36], Labrousse et al., 2002 [3], Rhydwen et al., 2002 [37], Malhotra et al., 2017 [29], Feneley et al., 1983 [19], Becker et al., 1999 [39], Crenshaw et al., 1999 [4], Menon et al., 2000 [21], Barker et al., 2003 [31], Pradhan et al., 2018 [15]. MI: myocardial infarction; VSD: Ventricular septal defect

**Figure 5:**
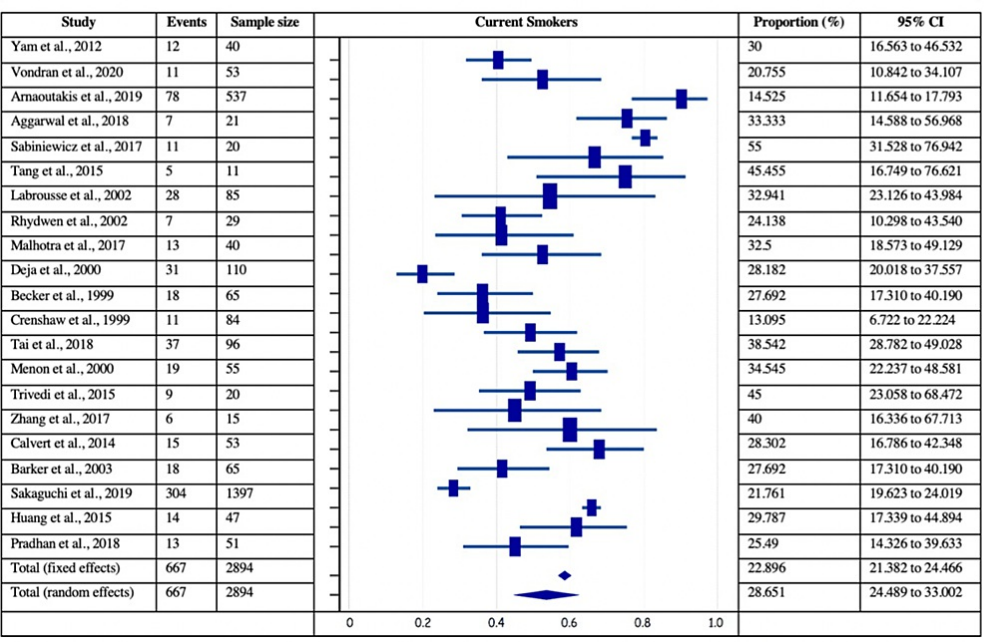
Forest plot showing smoking as a risk factor associated with post-MI VSD. Yam et al., 2012 [28], Vondran et al., 2020 [22], Arnaoutakis et al., 2019 [23], Aggarwal et al., 2018 [34], Sabiniewicz et al., 2017 [36], Tang et al., 2015 [1], Labrousse et al., 2002 [3], Rhydwen et al., 2002 [37], Malhotra et al., 2017 [29], Deja et al., 2000 [30], Becker et al., 1999 [39], Crenshaw et al., 1999 [4], Tai et al., 2018 [44], Menon et al., 2000 [21], Trivedi et al., 2015 [40], Zhang et al., 2017 [41], Calvert et al., 2014 [43], Barker et al., 2003 [31], Sakaguchi et al., 2019 [32], Huang et al., 2015 [5], Pradhan et al., 2018 [15]. MI: myocardial infarction; VSD: Ventricular septal defect

**Figure 6:**
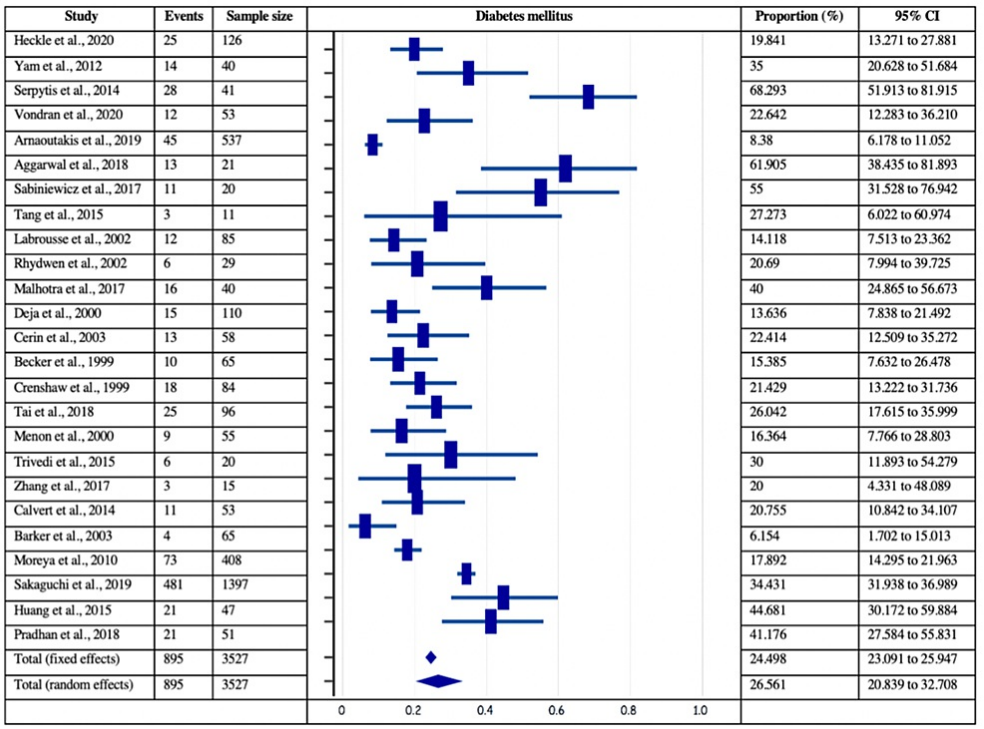
Forest plot showing diabetes mellitus as a risk factor associated with post-MI VSD. Heckle et al., 2020 [26], Yam et al., 2012 [28], Serpytis et al., 2014 [6], Vondran et al., 2020 [22], Arnaoutakis et al., 2019 [23], Aggarwal et al., 2018 [34], Sabiniewicz et al., 2017 [36], Tang et al., 2015 [1], Labrousse et al., 2002 [3], Rhydwen et al., 2002 [37], Malhotra et al., 2017 [29], Deja et al., 2000 [30], Cerin et al., 2003 [12], Becker et al., 1999 [39], Crenshaw et al., 1999 [4], Tai et al., 2018 [44], Menon et al., 2000 [21], Trivedi et al., 2015 [40], Zhang et al., 2017 [41], Calvert et al., 2014 [43], Barker et al., 2003 [31], Moreyra et al., 2010 [33], Sakaguchi et al., 2019 [32], Huang et al., 2015 [5], Pradhan et al., 2018 [15]. MI: myocardial infarction; VSD: Ventricular septal defect

**Figure 7:**
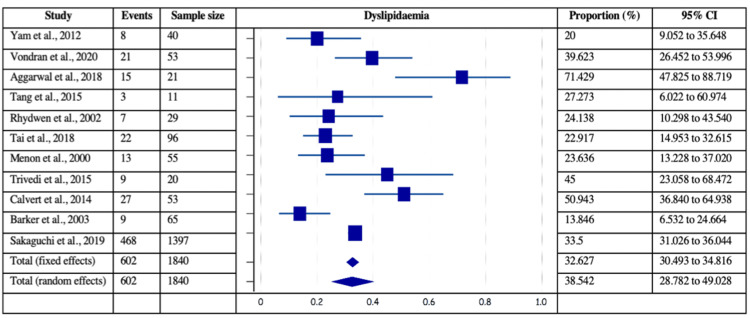
Forest plot showing dyslipidemia as a risk factor associated with post-MI VSD. Yam et al., 2012 [28], Vondran et al., 2020 [22], Aggarwal et al., 2018 [34], Tang et al., 2015 [1], Rhydwen et al., 2002 [37], Tai et al., 2018 [44], Menon et al., 2000 [21], Trivedi et al., 2015 [40], Calvert et al., 2014 [43], Barker et al., 2003 [31], Sakaguchi et al., 2019 [32]. MI: myocardial infarction; VSD: Ventricular septal defect

The risk factors of coronary artery disease, such as smoking, dyslipidemia, smoking, and prior MI, were analyzed for their independent estimated proportions with respect to patients with post-MI VSD. Among 40 included studies, the data was mentioned in 15 studies for prior MI (161/1324 patients), 25 studies for diabetes (895/3527 patients), 27 studies for hypertension (2074/3571 patients), 21 for current smoking (667/2894 patients), and 11 for dyslipidemia (602/1840 patients). Figures 4-7 delineate the forest plots and the estimated pooled proportion of associated risk factors of post-MI VSD. The data revealed a significant I2 index for all the risk factors, summarized in Table 2 (p<0.0001). Moreover, Egger’s and Begg's tests revealed no evidence of publication bias for any of the associated risk factors of post-MI VSD (p>0.05).

**Table 2:** Heterogeneity test and publication bias test values for risk factors. MI: myocardial infarction

Risk factor	I^2 ^index	p-value	95% CI	Egger’s test			Begg’s test	
				intercept	p-value	95% CI	Kendall’s Tau	p-value
Prior-MI	90.69%	<0.0001	86.34 to 93.65	2.85	0.132	-0.9781 to 6.6890	0.241	0.217
Diabetes mellitus	92.03%	<0.0001	89.45 to 93.98	0.18	0.88	-2.2918 to 2.6541	0.41	0.051
Smoking	94.86%	<0.0001	93.47 to 95.96	-1.9521	0.177	-4.8505 to 0.9463	0.17	0.22
Dyslipidaemia	78.65%	<0.0001	62.30 to 87.91	-0.1122	0.912	-2.3395 to 2.1151	0.257	0.27

Incidence of Cardiogenic Shock, Preoperative* IABP, and Concomitant CABG in Post-MI VSD Subjects*

**Figure 8:**
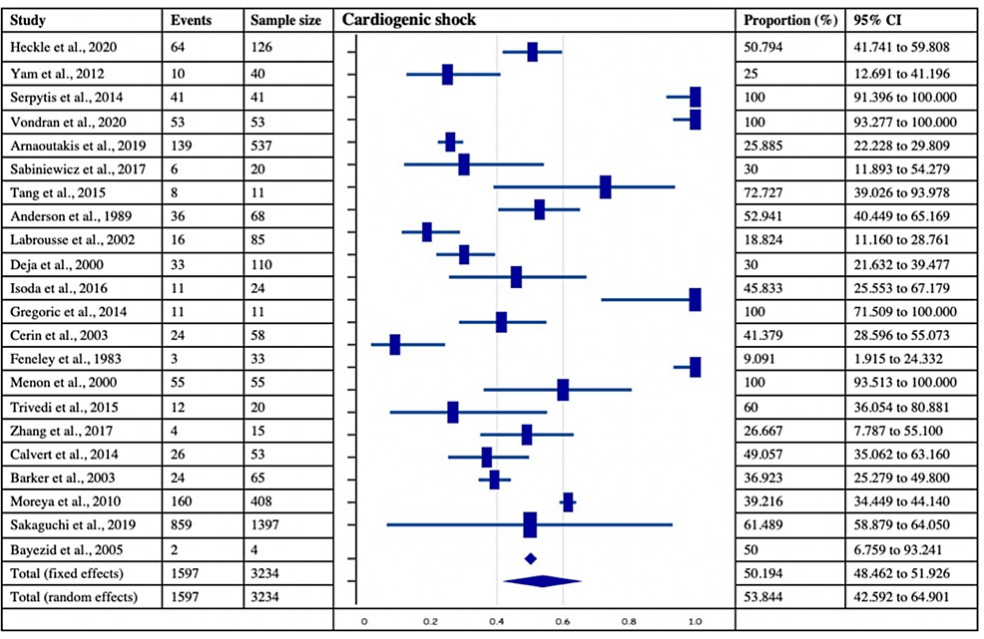
Forest plot showing the number of subjects with cardiogenic shock in post-MI VSD patients. Heckle et al., 2020 [26], Yam et al., 2012 [28], Serpytis et al., 2014 [6], Vondran et al., 2020 [22], Arnaoutakis et al., 2019 [23], Sabiniewicz et al., 2017 [36], Tang et al., 2015 [1], Anderson et al., 1989 [2], Labrousse et al., 2002 [3], Deja et al., 2000 [30], Isoda et al., 2016 [18], Gregoric et al., 2014 [11], Cerin et al., 2003 [12], Feneley et al., 1983 [19], Menon et al., 2000 [21], Trivedi et al., 2015 [40], Zhang et al., 2017 [41], Calvert et al., 2014 [43], Barker et al., 2003 [31], Moreyra et al., 2010 [33], Sakaguchi et al., 2019 [32], Bayezid et al., 2005 [10]. MI: myocardial infarction; VSD: Ventricular septal defect

**Figure 9:**
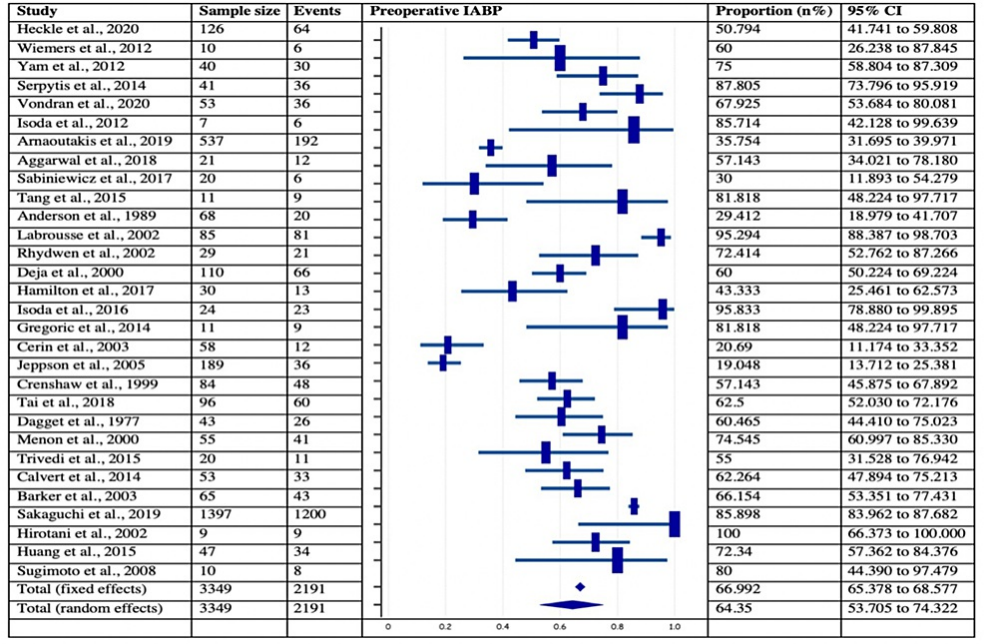
Forest plot showing the number of subjects with preoperative IABP in post-MI VSD patients Heckle et al., 2020 [26], Wiemers et al., 2012 [27], Yam et al., 2012 [28], Serpytis et al., 2014 [6], Vondran et al., 2020 [22], Isoda et al., 2012 [14], Arnaoutakis et al., 2019 [23], Aggarwal et al., 2018 [34], Sabiniewicz et al., 2017 [36], Tang et al., 2015 [1], Anderson et al., 1989 [2], Labrousse et al., 2002 [3], Rhydwen et al., 2002 [37], Deja et al., 2000 [30], Hamilton et al., 2017 [17], Isoda et al., 2016 [18], Gregoric et al., 2014 [11], Cerin et al., 2003 [12], Jeppson et al., 2005 [13], Crenshaw et al., 1999 [4], Tai et al., 2018 [44], Dagget et al., 1977 [7], Menon et al., 2000 [21], Trivedi et al., 2015 [40], Calvert et al., 2014 [43], Barker et al., 2003 [31], Sakaguchi et al., 2019 [32], Hirotani et al., 2002 [8], Huang et al., 2015 [5], Sugimoto et al., 2008 [42]. MI: myocardial infarction; VSD: Ventricular septal defect

**Figure 10:**
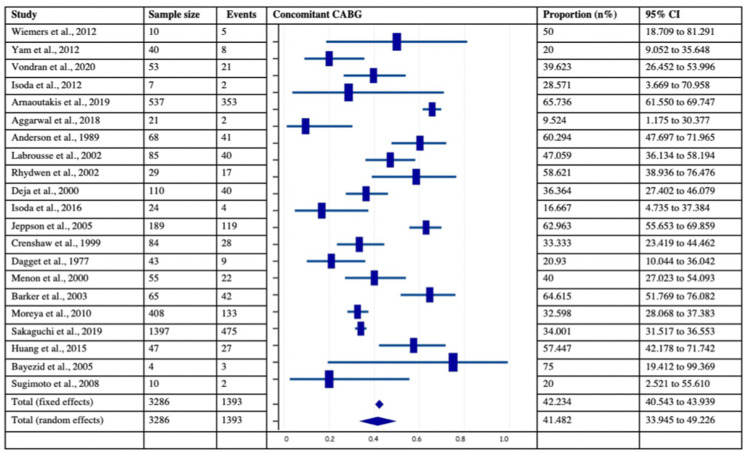
Forest plot showing the number of subjects with concomitant CABG in post-MI VSD patients Wiemers et al., 2012 [27], Yam et al., 2012 [28], Vondran et al., 2020 [22], Isoda et al., 2012 [14], Arnaoutakis et al., 2019 [23], Aggarwal et al., 2018 [34], Anderson et al., 1989 [2], Labrousse et al., 2002 [3], Rhydwen et al., 2002 [37], Deja et al., 2000 [30], Isoda et al., 2016 [18], Jeppson et al., 2005 [13], Crenshaw et al., 1999 [4], Dagget et al., 1977 [7], Menon et al., 2000 [21], Barker et al., 2003 [31], Moreyra et al., 2010 [33], Sakaguchi et al., 2019 [32], Huang et al., 2015 [5], Bayezid et al., 2005 [10], Sugimoto et al., 2008 [42]. MI: myocardial infarction; VSD: Ventricular septal defect; CABG: coronary artery bypass graft

Among 40 chosen studies with 4,028 patients, 22 studies with 3,234 patients, 30 studies with 3349 patients, and 21 studies with 3286 patients provided the data of cardiogenic shock, preoperative IABP, and concomitant CABG in Post-MI VSR subjects, respectively. The analysis revealed that the incidence of cardiogenic shock and preoperative IABP were observed in more than half of the post-MI VSR population with estimated pooled proportions of 50.19% (95% CI: 48.46-51.92%) and 66.99% (95% CI:65.37-68.57%) respectively. On the contrary, the pooled proportion of concomitant CABG was 42.23% (95% CI = 33.95 - 49.23). The forest plots of the respective variables are provided in Figures 8-10. Further, the I2 index of cardiogenic shock (I2 = 96.88 %, p<0.0001, 95% CI = 96.09-97.50), preoperative IABP (I2 = 96.86%, p<0.0001, 95% CI = 96.2- 97.41%), and concomitant CABG (I2 = 93.04%, p<0.0001, 95% CI = 90.65- 94.82%) were found to be significant. Lastly, Egger’s test and Begg’s test revealed no evidence of publication bias for the respective variables across selected studies.

Mortality as a Major Outcome

**Figure 11:**
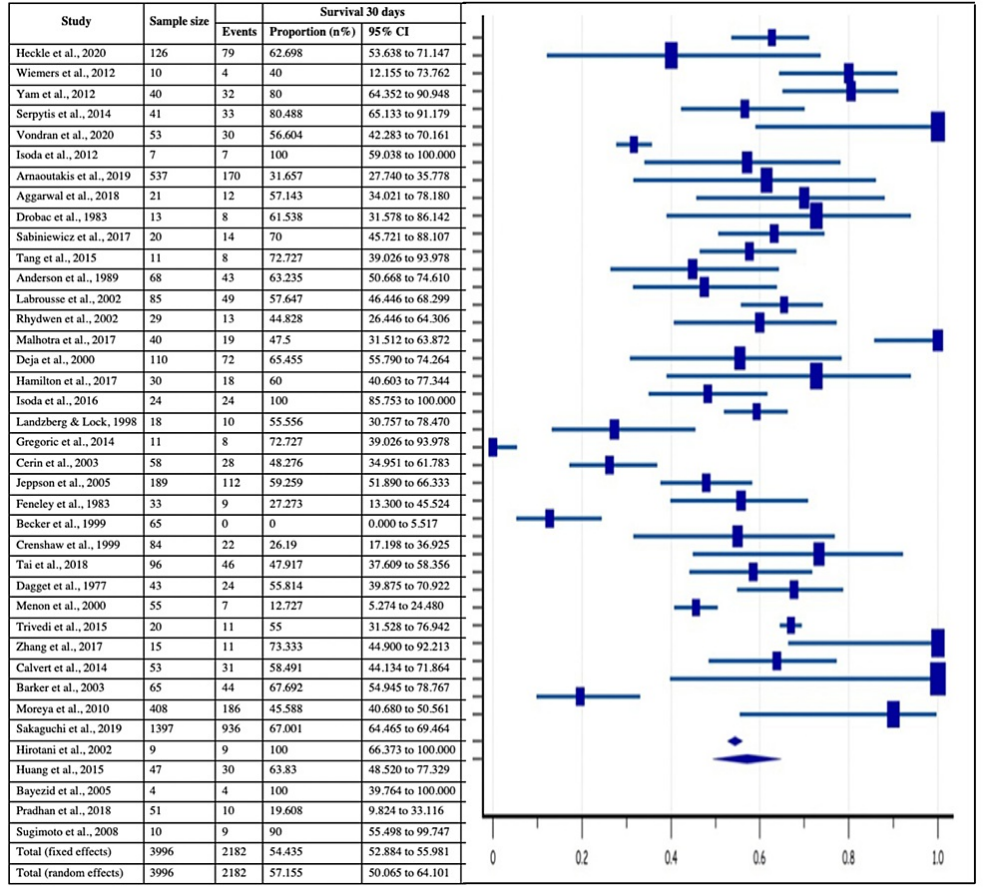
Forest plot showing 30 days survival in post-MI VSD patients Heckle et al., 2020 [26], Wiemers et al., 2012 [27], Yam et al., 2012 [28], Serpytis et al., 2014 [6], Vondran et al., 2020 [22], Isoda et al., 2012 [14], Arnaoutakis et al., 2019 [23], Aggarwal et al., 2018 [34], Drobac et al., 1983 [35], Sabiniewicz et al., 2017 [36], Tang et al., 2015 [1], Anderson et al., 1989 [2], Labrousse et al., 2002 [3], Rhydwen et al., 2002 [37], Malhotra et al., 2017 [29], Deja et al., 2000 [30] Hamilton et al., 2017 [17], Isoda et al., 2016 [18], Landzberg & Lock, 1998 [38], Gregoric et al., 2014 [11], Cerin et al., 2003 [12], Jeppson et al., 2005 [13], Feneley et al., 1983 [19], Becker et al., 1999 [39], Crenshaw et al., 1999 [4], Tai et al., 2018 [44], Dagget et al., 1977 [7], Menon et al., 2000 [21], Trivedi et al., 2015 [40], Zhang et al., 2017 [41], Calvert et al., 2014 [43], Barker et al., 2003 [31], Moreyra et al., 2010 [33], Sakaguchi et al., 2019 [32], Hirotani et al., 2002 [8], Huang et al., 2015 [5], Bayezid et al., 2005 [10], Pradhan et al., 2018 [15], Sugimoto et al., 2008 [42]. MI: myocardial infarction; VSD: Ventricular septal defect

**Figure 12:**
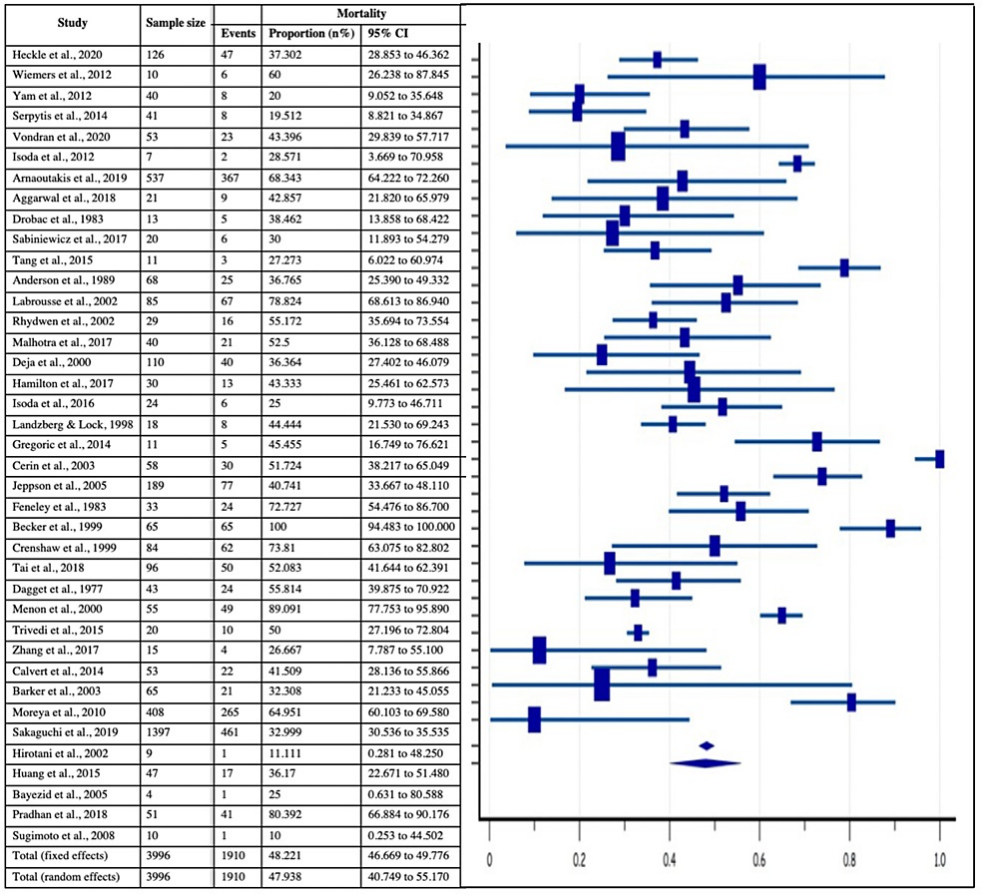
Forest plot showing mortality in post-MI VSD patients Heckle et al., 2020 [26], Wiemers et al., 2012 [27], Yam et al., 2012 [28], Serpytis et al., 2014 [6], Vondran et al., 2020 [22], Isoda et al., 2012 [14], Arnaoutakis et al., 2019 [23], Aggarwal et al., 2018 [34], Drobac et al., 1983 [35], Sabiniewicz et al., 2017 [36], Tang et al., 2015 [1], Anderson et al., 1989 [2], Labrousse et al., 2002 [3], Rhydwen et al., 2002 [37], Malhotra et al., 2017 [29], Deja et al., 2000 [30] Hamilton et al., 2017 [17], Isoda et al., 2016 [18], Landzberg & Lock, 1998 [38], Gregoric et al., 2014 [11], Cerin et al., 2003 [12], Jeppson et al., 2005 [13], Feneley et al., 1983 [19], Becker et al., 1999 [39], Crenshaw et al., 1999 [4], Tai et al., 2018 [44], Dagget et al., 1977 [7], Menon et al., 2000 [21], Trivedi et al., 2015 [40], Zhang et al., 2017 [41], Calvert et al., 2014 [43], Barker et al., 2003 [31], Moreyra et al., 2010 [33], Sakaguchi et al., 2019 [32], Hirotani et al., 2002 [8], Huang et al., 2015 [5], Bayezid et al., 2005 [10], Pradhan et al., 2018 [15], Sugimoto et al., 2008 [42]. MI: myocardial infarction; VSD: Ventricular septal defect

All the 40 included studies provided the data of 30-day survival and overall mortality associated with post-MI VSD, which was found to be 2182/3996 and 1910/3996 with their estimated pooled proportions of 54.43% (95% CI: 52.88-55.98%) and 48.22% (95% CI: 4-12.3%)respectively, as shown in Figure 11-12. Moreover, heterogeneity tests of 30-day survival and overall mortality revealed significant inconsistency with I2 index of 93.90% (p<0.0001, 95% CI = 92.5-95.03) and 94.13% (p<0.0001, 95% CI = 92.81-95.21).

**Figure 13:**
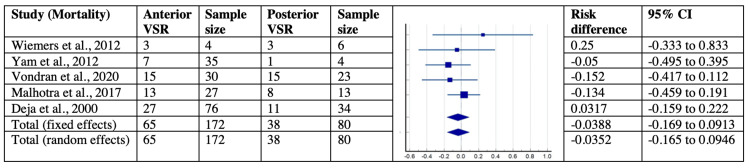
Forest plot showing mortality with respect to location of VSR in post-MI VSD patients. Wiemers et al., 2012 [27], Yam et al., 2012 [28],  Vondran et al., 2020 [22], Malhotra et al., 2017 [29], Deja et al., 2000 [30]. MI: myocardial infarction; VSD: Ventricular septal defect; VSR: ventricular septal rupture

**Figure 14:**
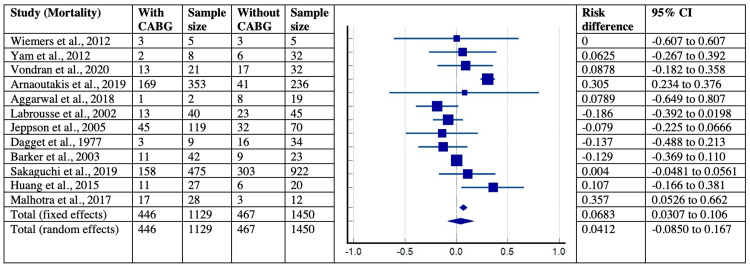
Forest plot showing mortality with respect to concomitant CABG in post-MI VSD patients. Wiemers et al., 2012 [27], Yam et al., 2012 [28], Vondran et al., 2020 [22], Arnaoutakis et al., 2019 [23], Aggarwal et al., 2018 [34], Labrousse et al., 2002 [3], Jeppson et al., 2005 [13], Dagget et al., 1977 [7], Barker et al., 2003 [31], Sakaguchi et al., 2019 [32], Huang et al., 2015 [5], Malhotra et al., 2017 [29]. MI: myocardial infarction; VSD: Ventricular septal defect; CABG: coronary artery bypass graft

Furthermore, the mortality concerning the location of VSR and concomitant CABG was corroborated in five studies with 252 patients and 12 studies with 1129 patients, respectively. The risk difference in mortality concerning the location of VSR and concomitant CABG was found to be -0.039 (95% CI = -0.169-0.091) and 0.068 (95% CI = 0.031-0.11) with their significant I2index of 96.88 % (p<0.0001, 95% CI = 96.09-97.50) and 96.86 % (p<0.0001, 95% CI = 96.2- 97.41%). Lastly, the publication bias using Egger’s and Begg’s tests suggested no evidence of asymmetry and small-study effects for all the analyzed variables in post-MI VSD patients across selected studies. The forest plots for mortality concerning the location of VSR and concomitant CABG are shown in Figures 13-14.

Discussion

Post-MI VSR poses a major clinical challenge due to high mortality [1-3]. Several studies in the literature have focussed on the surgical outcomes of the disease; however, there is paucity in the current knowledge concerning risk factors and associated factors. Based on this background, the present systematic review and meta-analysis attempt to pool the overall effect of different risk factors and associated clinical outcomes in post-MI VSD patients.

In the era of surgical interventions in patients with post-MI VSD, the incidence of post-MI VSD has significantly been reduced [3]. Evidence in the literature reported a 19.66% early mortality rate in post-MI VSD patients following VSD closure [15]. In our study, the pooled proportion of 30-day survival was estimated to be 54.43%, signifying the upper edge of the defined mortality range. Further, several guidelines recommend urgent and early surgical interventions to manage post-MI VSD [17]. The available data reported the average timing of MI to VSD diagnosis as 4.5 ± 3.3 days and the average timing from VSD diagnosis to repair as 21.5 ± 40.1 days. Concerning this period, early mortality falls 45-50%, similar to overall mortality.

In most cases, delay in the surgical intervention occurs because surgeons wait for the formation and improvement of tissue scars [18-20]. Early repair is needed in case of hemodynamic compromise, on high inotrope, and evidence of reduced tissue perfusion like increasing urea creatinine and liver enzymes. Otherwise, we can wait for some days. The operative outcome is better if operated after the acute episode provided good hemodynamics on minimal inotrope. Therefore, we suggest that average timing from VSD diagnosis to repair should be considered an important factor that adversely affects survival rates. Current guidelines recommend immediate surgical VSD closure, irrespective of the patient's hemodynamic status. However, surgery in older patients and patients with poor right ventricular function is futile because mortality approaches 100%.

Accumulating evidence in the literature suggests the association of various risk factors of coronary artery disease, such as smoking, dyslipidemia, smoking, and prior MI, with the development of post-MI VSD [3-6]. The pooled proportion of prior MI, diabetes, smoking, and dyslipidemia was estimated to be 10.76%, 24.49%, 22.89%, and 32.62%, respectively. Due to insufficient data, we failed to link the mentioned variables as risk factors of post-MI VSD. Expert consensus and data in the literature report the association of several operative mortality risk factors, such as cardiogenic shock and pre-procedure IABP usage, with post-MI VSD [21-22]. Our study analyzed the data for the pooled proportion of cardiogenic shock and pre-procedure IABP usage in patients. The incidence of cardiogenic shock and preoperative IABP were observed in more than half of the post-MI VSD population with estimated pooled proportions of 50.19% (95% CI: 48.46-51.92%) and 66.99% (95% CI: 65.37-68.57%) respectively. Arnaoutakis et al. collected the data from the Society of Thoracic Surgeons (STS) national database concordantly with our data. The authors reported the incidence of cardiogenic shock in 51.7% of the patients at the time of admission or intervention, and 30-day mortality was 42.9% 23. In two different studies, Thiele et al. and Egbe et al. identified and considered cardiogenic shock as an important predictor of 30-day mortality [24-25]. However, we failed to establish the link between these risk factors and mortality rate due to lack of data. 

The localization of MI or VSR may be other considerable variables to elude worse outcomes. In the present data, we observed that the number of patients with anterior localization of disease was significantly higher than those with posterior wall or septum defects, signifying that the anterior wall or septum is more vulnerable than the posterior wall for both MI and VSR. Moreover, the risk difference in anterior vs. posterior location of both MI and VSR was found to be statistically significant. Furthermore, the mortality concerning the location of VSR was also assessed, and the risk difference in mortality with anterior or posterior localization of VSR was found to be statistically non-significant. The postoperative course is stormier in posterior VSR due to associated right ventricular dysfunction. The data suggest that the location of the defect did not influence the survival rate of patients with anterior or posterior defects. However, only 5 studies with 252 patients corroborated this data [26-30]. More studies are needed to predict the relationship between survival rate and the location of the defect.

Furthermore, multi-vessel coronary artery disease patients underwent CABG during VSD repair [31-35]. Substantial evidence suggests that concomitant CABG provides no survival advantage to patients with post-MI VSD36-42. Thus, concomitant CABG remains a controversial subject in the surgical management of post-MI VSD. In our study, the pooled proportion of concomitant CABG was found to be 42.23% (95% CI = 33.95 - 49.23), suggesting little less than half of the patients underwent CABG at the time of VSD repair. We further assessed mortality in patients who received concomitant CABG at the time of VSD repair. The risk difference in patients with CABG and without CABG was statistically significant, suggesting the association concomitant CABG with mortality. 

Limitations

The study has a few limitations. Firstly, the identified studies lacked information on the intervention techniques for the surgical management of patients with post-MI VSD, which is a major drawback. Secondly, no sufficient data was given on the interval between VSD diagnosis to repair and the location of MI in association with mortality.

## Conclusions

The present systematic review and meta-analysis evaluated the surgical outcomes and associated risks in the patients of post-MI VSD. A statistically significant risk difference was noted in the patients' anterior vs. posterior locations of MI and VSD. The 30-day survival rate remained low, while the mortality rate was higher even after better surgical interventions for the management of post-MI VSD. Our data suggest that concomitant CABG is an additional factor corroborating to mortality. We conclude that time from VSR diagnoses to repair should be considered for the timely management of patients with post-MI VSD that may provide better survival benefits.
